# A89 LIVER TRANSPLANT REFERRAL PATTERNS FROM THE ATLANTIC CANADIAN PROVINCES

**DOI:** 10.1093/jcag/gwad061.089

**Published:** 2024-02-14

**Authors:** C Harper, S Allen, J Zhu

**Affiliations:** Internal Medicine, Dalhousie University, Halifax, NS, Canada; Nova Scotia Health Authority, Halifax, NS, Canada; Nova Scotia Health Authority, Halifax, NS, Canada

## Abstract

**Background:**

End-stage liver disease (ESLD) is one of the leading causes of deaths in Canada. Liver transplantation (LT) is the ultimate treatment option for patients with ESLD. A variety of factors may influence patient outcomes before a liver transplant is offered. There have been no studies looking at the characteristics of patients referred for LT in Atlantic Canada at the prelisting and wait-listing stages.

**Aims:**

To examine various patient characteristics, including age, sex, MELD score, BMI, rural vs. urban residency, and diagnosis that may be important to the LT work up process. To better understand referral patterns and potential barriers to LT in Atlantic Canada including geographic factors, the COVID-19 pandemic, and the evolution of underlying liver disease.

**Methods:**

This is a retrospective cohort study using Multiorgan Transplant (MOTP) database to identify all active referrals sent to the Atlantic liver transplant program from four Atlantic Canadian provinces between January 1, 2017 and September 30, 2023.

**Results:**

There were 533 LT referrals to the MOTP in the observation period. From the listed patients (217), 60% were transplanted and 25% died on the waiting list. Of the patients that did not progress to being listed (56%), 197 (37%) of the referrals were withdrawn and 99 (18.6%) died prior to being listed.

There was no significant difference in mean BMI, age, listing MELD-Na, or diagnosis (p=0.35) among active referrals between pre-COVID-19 (Jan.1 2017-Mar.30 2020) and COVID-19 periods (Apr.1 2020-Sept.30 2023). There was 6% reduction in referral number from all Atlantic provinces in COVID vs. pre-COVID period, and 25% reduction in referrals for hepatocellular carcinoma (HCC) in COVID.

The ratio of patients ampersand:003C 65 yrs to ampersand:003E/= 65 yrs decreased during COVID (2.88) compared to pre-COVID (3.57). For pre and post COVID period, there were similar numbers of patients withdrawn/died prior to listing (54.7%, 54.8%). There were less patients listed (43.0%, 38.2%) and a decrease in patients transplanted (32%, 28%) pre and post COVID.

We analyzed the number of referrals from urban and rural areas. Overall, 33% of referrals were urban and 67% of referrals were rural based on patient residence. The ratio of rural to urban area referrals was 1.80 pre COVID and 2.24 during Covid, indicating an increasing number of referrals from rural centers post-COVID.

**Conclusions:**

Although there was a robust number of LT referrals during the pandemic, there was a decrease in number of referrals and transplants after COVID. There is a notable decrease in referrals for HCC after COVID. One plausible reason is reduced HCC surveillance during the pandemic. There was an increase in referrals from rural areas during COVID. Further analysis will be needed to determine any factors that affect LT referral patterns., with the goal of identifying reasons for this change and making appropriate recommendations to the MOTP.

**Funding Agencies:**

None

